# Saccadic reaction time in mirror image sectors across horizontal meridian in eye movement perimetry

**DOI:** 10.1038/s41598-021-81762-y

**Published:** 2021-01-29

**Authors:** Deepmala Mazumdar, Najiya S. Kadavath Meethal, Ronnie George, Johan J. M. Pel

**Affiliations:** 1 Vestibular and Ocular Motor Research Group, Department of Neuroscience, Erasmus MC, Rotterdam, The Netherlands; 2 Medical and Vision Research Foundation, Chennai, India; 3 Department of Neuroscience, Room EE 1453, Erasmus MC, PO Box 2040, 3000 CA Rotterdam, The Netherlands

**Keywords:** Medical research, Translational research

## Abstract

In eye movement perimetry (EMP), the saccadic reaction time (SRT) to ‘seen’ visual stimuli are delayed in glaucoma. Evaluating SRT behaviour in hemi-field sectors could refine its clinical implication. The development phase included 60 controls retrospectively and for the test cohort in evaluation phase, another 30 healthy subjects and 30 glaucoma patients were recruited prospectively. The SRTs were used to calculate the normative limits within 5 predefined hemi-field sectors. Scores were assigned to probabilities for SRT at the level of 5%, 2.5% 1% and 0.5%. Per sector pair, a probability score limit (PSL) was calculated at each of the four levels and were compared with the scores obtained from the test cohort. The classification accuracy ‘normal versus abnormal’ was assessed for PSL in EMP and compared with glaucoma hemi-field test in standard automated perimetry. We found no statistically significant differences in SRTs between the mirror sectors in healthy subjects. The PSL at 2.5% had moderate classification accuracy with a specificity of 77% and sensitivity 70%. This could be suggestive of an SRT delay in the overall visual field in glaucoma.

## Introduction

Standard automated perimetry (SAP) is the most widely used and accepted functional test to determine the extent of the visual field. SAP uses differential light sensitivity threshold values and it has been an integral part of the diagnosis and management of glaucoma^1,2^. eye movement perimetry (EMP) on the other hand is an unconventional approach to assess the extent of the visual field using saccadic eye movements (SEM). This technique of testing the visual field relies on reflexive SEM responses from a central fixation point presented in the middle of the screen to consecutively shown stimuli in the periphery. From all the goal-directed SEMs to the seen peripheral stimuli, the saccadic reaction time (SRT) is quantified. Here, a peripheral stimulus is labelled as seen when a SEM is initiated in the direction of the peripheral stimulus and covers > 50% of the path^3–6^. Therefore, this method introduces the possibility to plot all obtained SRT values as a function of the tested locations as well as a measure of subject’s visual field responsiveness. It was demonstrated that analogous to light sensitivity threshold, SRT depends on subjects’ age, and factors in tested visual field such as stimulus intensity and eccentricity^3,6,7^. Moreover, the SRTs were found to be significantly delayed in glaucoma patients when compared to their age-matched healthy subjects^8–12^. In addition, the physiological variability of SRT was considered and empirical probability plots were created to display the presence and extent of the defect along with the statistical significance^13^. These plots can aid the interpretation of visual field report produced by EMP.

In the Humphrey field analyser (HFA), global indices such as mean deviation (MD), pattern deviation (PD) are sensitive to the reduction in light sensitivity as assessed in a population with healthy eyes. Since, glaucomatous visual field defect was shown to be localised and asymmetrical across the horizontal meridian, methods comparing the hemi-fields across the horizontal meridian for additional diagnostic purpose have been reported in the literature^14,15^. To differentiate between typical glaucomatous defects from the diffuse loss in light sensitivity [caused by media opacities], further refinements of the basic approach were made and termed as glaucomatous hemi-field test (GHT). This modified approach, introducing new sector borders corresponding to normal nerve fibre layer arrangements, has been described by Asman and Heijl^15^. The GHT comprises of 10 sectors [5 horizontal hemi-field sector pairs], where the superior hemi-field sectors are the mirror images of the inferior hemi-field.

Though SRT was found to be delayed in glaucoma, the nature of the glaucomatous visual field defect assessed by SRT is still unexplored. Since the construction of GHT sectors were based on retinal nerve fibre layer arrangements, the exploration of SRT behaviour in GHT sectors may add to a more concise approach of interpreting the EMP reports with respect to the glaucomatous visual field defect. In contemplation of investigating the behaviour of SRT in the GHT sectors, we first aimed to evaluate the SRT behaviour in the 5 superior and 5 mirror inferior GHT sectors in healthy eyes. Next, the classification accuracy of EMP based on sector wise comparison of SRTs within the hemi-fields was calculated in an additional group of healthy and glaucomatous eyes.

## Materials and methods

This study comprised of two phases. The first phase is the development of hemi-field sectors normative data. Here, estimation of point-wise normative limits based on SRT values are calculated for each of the five hemi-field sectors in healthy eyes. The second phase is the evaluation of its classification accuracy in a new set of healthy eyes and glaucomatous eyes.

### Development of hemi-field sectors normative data

#### Participants

Data obtained in the right eye of sixty healthy subjects between 20 and 70 years of age were (randomly) selected from our database to estimate the normative limit for each sector of the Hemi-field. These data were collected between 2012 and 2015 and part of this data have been published^3^. The 60 eyes were grouped into five age groups: 20–29 years, 30–39 years, 40–49 years, 50–59 years and 60 years and above. Each subject had the measurement data available from the Humphrey field analyser (HFA) II 750 (Carl Zeiss Meditec Inc, Dublin, CA, USA), program 24-2, with Swedish interactive threshold algorithm (SITA) standard strategy and EMP. The test grids (co-ordinates) in both methods were identical^3^.

##### Identifying glaucoma hemi-field sectors in EMP

**Figure 1:**
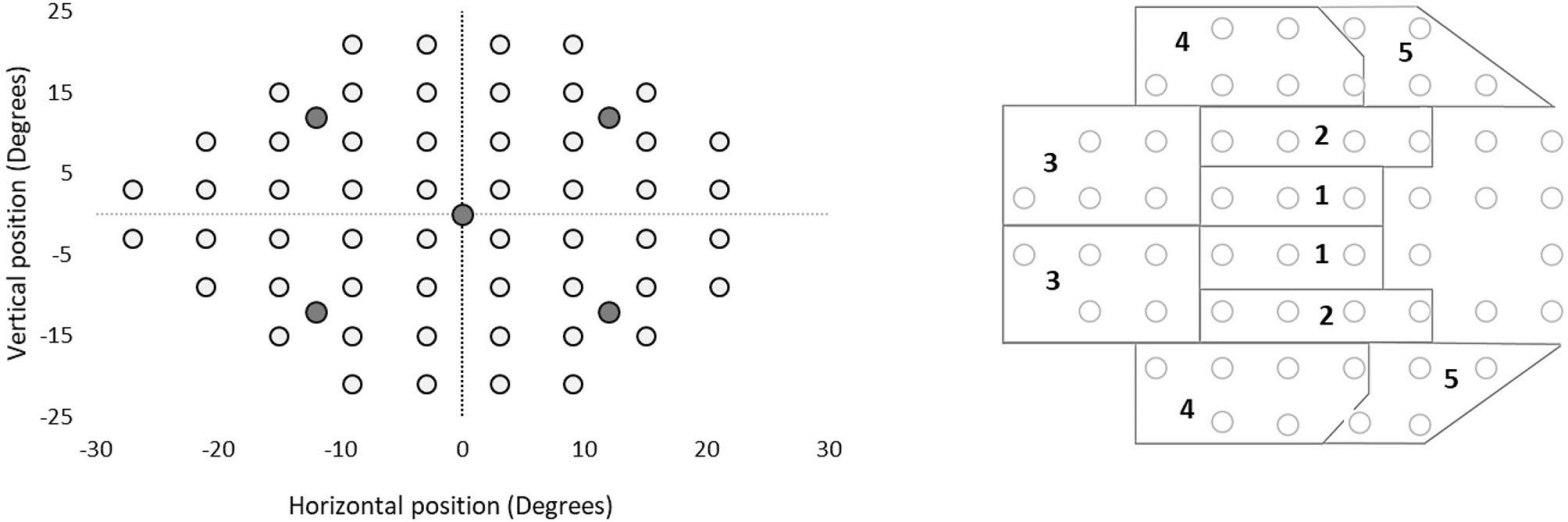
The glaucoma hemi-filed sectors in EMP test grid; Left panel: EMP 54 points test grid (depicted as circles) with the stimulus locations (blank circles) in degrees and illustrating the position of the fixation stimuli in filled circles (grey), Right panel: EMP test grid partitioned into 10 sectors corresponding to retinal nerve fibre layer anatomy [Åsman and Heijl (1992)]. The 10 sectors include 5 demarcated sectors in the superior hemi-field and five mirrored sectors in the inferior hemi-field across the horizontal meridian. The five sector pairs are numbered from 1 to 5.

Analogous to 24-2 SITA Standard GHT sectors, ten sectors were identified and composed of five sectors in the superior hemi-field and their mirror images in the inferior hemi-field, see Fig. 1.

##### Estimation of normative limits

Limits of normality were estimated from a previously published EMP database, where SRT interaction was evaluated for different factors (age, stimulus intensity and eccentricity) within the tested visual field in healthy subjects^3^. The SRT interaction implies to how different factors such as age of the participant, presented stimulus intensity and eccentricity of the peripheral stimulus affect SRT in the healthy individuals in the EMP test. To estimate the likelihood of SRT for a specified age group, the probability for SRT at the level of 5%, 2.5%, 1% and 0.5% were calculated from this dataset^13^. The normal limits of the SRT were calculated based on the empirically determined deviations from the age-corrected normal SRT. Thus, the 5%, 2.5%, 1%, 0.5% significance levels of SRT distributions were determined separately for each tested point in the visual field in each age group calculating the inverse of the cumulative standardised normal distribution. Any SRT outside the 5% percentile limit were flagged, which means that only 5% of the healthy population of that age group might exhibit such SRT value, whereas the other 95% would show faster SRTs.

#### Assigning probability score (PS) for delayed SRT

The SRTs obtained at all tested locations were checked if they were depressed to a probability level that was adapted from the point—by- point probability scores of Åsman and Heijl^15^. In the healthy subjects, the raw SRT values were compared with the age matched normative reference. Higher scores were assigned to the locations showing extreme delays in SRT compared with the age corrected normal reference database. Instead of using the raw SRT values, these score were used to calculate the normative limits for each of the 10 hemi-field sectors. A probability score (PS) was assigned against the probability levels, i.e., non-significant—PS = 0, 5%—PS = 1, 2.5%—PS = 2 & 1%—PS = 5. For the locations with extreme delay in SRT at the level of 0.5%, an individualised score was assigned based on the age corrected limit which corrected for the height of normal reference visual field. This adjusted the overall ceiling effect of SRT delay in the visual field. Here, the scoring was based on the significant delay in SRT from an age matched normal value shown in EMP [PS = 10 × Delay in SRT (milliseconds)/0.5% (SRT limit)], see Table 1.

**Table 1:** The probability score (PS) assigned for SRT delayed at each probability levels.

Probability scores	
The significant probability level for delay in SRT	Probability score
Not significant	0
5%	1
2.5%	2
1%	5
0.5%	$\frac{{{1}0 \times {\text{Delay}}\,{\text{in}}\,{\text{SRT}}\,\left( {{\text{milliseconds}}} \right)^{{\text{a}}} }}{{0.{5}\% \, \left( {{\text{SRT}}\,{\text{limit}}} \right)^{{\text{b}}} }}$

^a^Delay in SRT is the residual SRT after subtracting the SRT raw value from the age matched normal reference value for that particular location.

^b^SRT limit is the expected age corrected SRT value for that location at the probability level of 0.5%. SRT indicates saccadic reaction time in milliseconds.

#### Determination of probability score limits (PSL) for hemi-field sectors

To estimate the limits for the hemi-field sectors, the PS of all points in each of the 10 sectors were calculated. The absolute difference between PS_upper _− PS_lower_ was calculated for each sector mirror image pair in 60 healthy eyes. This resulted in 5 probability score limits (PSL) for each sector pair. This procedure was repeated to obtain the PSLs at the level of 5%, 2.5%, 1% and 0.5%, see Table 2. Later these values were used to estimate the diagnostic accuracy of PSLs at different levels (see, Evaluation of SRT in hemi-field sectors results).

**Table 2:** The normative probability score limits (PSL) in four levels were determined for each hemi-field sector pair from the absolute up-down differences in probability scores obtained from the SRT using eye movement perimetry.

Probability score limits (PSL)				
Hemi-field sector pair	Levels (%)			
	5%	2.5%	1%	0.5%
1	14	16	18	19
2	12	14	15	17
3	17	19	21	23
4	24	26	29	32
5	19	21	24	26

SRT, saccadic reaction time.

### Evaluation of SRT in hemi-field sectors

#### Participants

A new group of thirty healthy subjects and thirty glaucoma patients between 20 and 70 years of age were recruited from the day to day glaucoma clinic in Sankara Nethralaya, Chennai, India. The post hoc power analysis for the sample size of the phase that dealt with evaluation of SRT in hemi-field sectors revealed the power of 100% with an alpha error 0.05. The age of the subjects included here is representative of the populations reported in clinic for mild and moderate glaucoma cases. These participants were recruited to estimate the classification ability of the modified Glaucomatous Hemi-field based on SRT values. Subjects with spherical ametropia greater than ± 5.00 DSph and cylindrical ametropia of more than -2.00 DSph, best corrected Visual Acuity less than 20/40, 0.8 M and ophthalmic conditions that are known to affect eye tracking, such as ptosis, corneal opacity and oculomotor nerve palsy, manifested strabismus with deviation > 6 prism dioptre, presence of nystagmus, any history of impaired cognitive status, mental illness or neurological disorders were excluded. Healthy subjects were defined as those with an Intra Ocular Pressure (IOP) less than 21 mmHg, with no family history of glaucoma or any other ocular pathologies, a healthy anterior and posterior segment along with a normal visual field. The visual field was assessed using the 24-2 Humphrey visual field analyser (HFA) consisting of 54 points and the Eye Movement Perimeter setup using the same 54 point test grid in random order. The subjects, who were unable to perform SAP reliably, were not included in the study. Reliability criteria for HFA tests included fixation loss, < 20%; false positive, < 15% according to the recommendations of the manufacturer. Subjects with primary glaucoma were defined according to the definition and classification by Foster et al*.* This classification was used to discriminate between healthy and glaucoma and was used as reference for further comparisons^16^. The disease severity of the glaucoma patients were classified into normal, mild and moderate glaucoma using the SAP visual field reports based on Hodapp, Parrish and Anderson’s (HAP) classification^17^. The eligible subjects were informed about the test and written informed consent was obtained prior to the clinical examination. The study was approved by institutional review board and Ethics committee of Vision Research Foundation, Chennai, India. The study adhered to the Declaration of Helsinki for research involving human subjects^18^.

#### Eye movement perimetry (EMP)

The EMP measurement setup has been previously described^3,10–13^. Briefly, the test setting includes a 17″ Thin Film Transistor (TFT) display with an inbuilt Tobii 120 eye tracking device of refresh rate 120 Hz with an accuracy of 0.5° at a testing distance of 60 cm. The test started with a nine point calibration test provided in the eye tracker Software Development Kit inbuilt in the Tobii 120, where the subject needs to follow a red circular target and the calibration was repeated for the locations that had insufficient gaze data sample. If one or more points were not correctly calibrated, these points were re-calibrated until all points met the criteria (within 0.5 deg. spatial accuracy). Only in a few cases in both healthy and glaucoma group, a re-calibration was needed irrespective of the subject group due to positional alignment or unable to comprehend the instruction in the first time. The test was performed under monocular viewing conditions by covering the non-tested eye with a black polymethyl methacrylate plate (PMMA), which permitted the passage of infrared light allowing stable binocular gaze tracking.

In EMP, stimuli with intensity of 214 cd/m^2^ were presented at 54 locations against a background of 152 cd/m^2^. The test started with a fixation point presented at the centre of the screen, i.e. position (0°, 0°). After a consistent fixation of 0.5 s, a peripheral stimulus was presented for a maximum duration of 1200 ms (ms) using an overlap paradigm. Upon detection, each subject was encouraged to fixate this peripheral stimulus. After a fixation duration of 200 ms, this stimulus disappeared and the subject re-fixated the central fixation point to repeat this sequence. In total, 54 peripheral stimuli were shown in a consecutive manner using the same visual field test co-ordinates used in the 24-2 SITA standard of HFA. To warrant a visual angle of ~ 60° horizontally and ~ 45° vertically, we altered the central fixation position (0°, 0°). When 14 peripheral stimuli had been presented, the central fixation stimulus was shifted to an eccentric position (− 12°, − 12°). Here, 10 peripheral stimuli were shown and this was repeated another three times (− 12°, 12°), (12°, 12°) and (12°, − 12°)], see also Fig. 1, left panel for an illustration of the 5 different fixation points used. On average, the test duration was ~ 7–8 min per eye.

The trajectory and time course of each Saccadic Eye Movement (SEM) towards a peripheral stimulus was first visually inspected and next analysed using a previously published decision algorithm developed in Matlab Version 7.11 (Math Works, Natick, MA, USA)^3,11,12^. A peripheral stimulus was labelled as ‘seen’ if the responses adhered to the following criteria: (a) A SEM, initiated towards the presented visual stimulus, (b) SEM, at onset, was in the direction of the peripheral stimulus and covered > 50% of the total fixation to peripheral stimulus distance, (c) The angular disparity of less than 45° between the direction of the primary SEM and the peripheral stimulus location. A stimulus was labelled as ‘unseen’ if the above criteria were not satisfied. ‘Invalid’ responses were labelled when eye movement data was not available due to blinking or failure in pupil detection and was excluded from the analysis. An EMP report with more than 25% of invalid responses was considered as ‘unreliable’. SRT was defined as the time difference between the stimulus presentation and the onset of the SEM towards the direction of the peripheral stimulus based on the gaze velocity criterion by calculation the reaction time at which the eye velocity crossed 80°/seconds.

#### Data preparation

For each subject, the SAP 24-2 SITA standard test report was collected with a GHT notification ‘*with in normal limits’* and ‘*outside normal limits*’. From the EMP measurement, the PSL was determined for each hemi-field sector pair for each subject of the test cohort belonging to the evaluation of SRT in hemi-field sectors. The PSL’s of each subject was compared with the normal limits of PSL obtained in the controls. A visual field of a subject was classified as abnormal if the PSL of one or more of the five sector pairs were found outside the normal limits for PSL. This was calculated for all four levels (5%, 2.5%, 1% and 0.5%) of PSL.

#### Statistical analysis

The right eye gaze data of each participant obtained at stimulus intensity 214 cd/m^2^ was considered for analysis. Statistical analysis were performed using SPSS (Statistical Package for Social Sciences, Version 15, Chicago, IL, USA). Assumptions of normality was assessed using Kolmogorov–Smirnov test.

A descriptive analysis of the demographic details were done for both the phases. The Kolmogorov–Smirnov test was used to assess the normality assumptions of the quantitative variable and appropriate parametric tests were chosen. Type I error was kept at 5% level. A two-tailed independent t test was used for comparison between the groups. Factorial ANOVA was used assess the SRT behaviour in different hemi-field sectors in healthy subjects of different age groups; a significant interaction was interpreted by a subsequent post-hoc Student–Newman–Keuls test (SNK). Receiver Operating Characteristic (ROC) curves were plotted to evaluate the diagnostic ability of the 4 sets of limits (5%, 2.5%, 1% and 0.5%) using the PSLs obtained from the development phase (see Table 2). The Area Under the Curve (AUC) values were considered as a measure to quantify the diagnostic accuracy of PSL. The subjects were first divided into healthy and glaucoma using Foster et al. classification^16^. Next, in order to evaluate the ability of PSL to detect glaucomatous visual field defects the glaucoma subjects were divided based on disease severity into mild and moderate glaucoma based on HAP criteria^17^ of Standard Automated Perimetry. The number of subjects were represented in 2 × 2 contingency tables. The sensitivity and specificity were calculated based on the cell frequencies observed in each category keeping the Foster et al.^16^ and HAP^17^ classification as standard reference.

## Results

A total of 90 healthy subjects and 30 glaucoma patients aged between 20 and 70 years were recruited in the study. Table 3 presents the demographic details [mean (SD)] and the summary of the data. The glaucoma group was divided into mild and moderate glaucoma with 15 patients in each group using HAP criteria^17^ (Table 4).

**Table 3:** Demographics and data summary of the development of hemi-field sectors normative data in EMP and evaluation of SRT in hemi-field sectors study population.

	Development of hemi-field sectors normative data		Evaluation of SRT in hemi-field sectors		
	Healthy (n = 60)	*p* value	Healthy (n = 30)	Glaucoma (n = 30)	*p* value
Age (years)	44 (13)	0.94*	45 (13)	53 (13)	< 0.001^†^
Gender (%)	Male 53%	0.47^¤^	Male 57%	Male 79%	< 0.001^‡^
IOP (mmHg)	16 (3)	0.10*	15 (3)	15 (6)	0.95^†^
Cup-disc ratio (–)	0.5 (0.10)	0.21*	0.5 (0.14)	0.7 (0.2)	< 0.001^†^
MD (dB)	− 1.5 (1.5)	0.40*	− 1.8 (1.5)	− 8.4 (4.5)	< 0.001^†^
SRT (ms)	402 (40)	0.20*	380 (35)	615 (56)	< 0.001^†^

Data expressed as mean (SD); Type I error was kept at 5% level and two-tailed tests were used.

IOP, intra-ocular pressure in mmHg; MD, mean deviation from standard automated perimetry in decibel (dB); SRT, saccadic reaction time from eye movement perimetry in milliseconds (ms).

*Independent T-test between healthy eye from development and evaluation phase.

^¤^Chi-square test for proportion of male and female between the groups.

^†^Independent T test for the evaluation phase of the study population of normal and glaucoma 30 in each group.

^‡^Chi-square test for proportion of male and female between the groups.

**Table 4:** Demographics and data summary of the glaucoma group from Evaluation of SRT in hemi-field sectors phase study population.

	Mild glaucoma (n = 15)	Moderate glaucoma (n = 15)	*p* value*
Age (years)	51 (12)	57 (11)	0.22
IOP (mmHg)	14 (5)	16 (4)	0.95
Cup-disc ratio (–)	0.5 (0.2)	0.8 (0.1)	< 0.001
MD (dB)	− 3.5 (2.4)	− 9.5 (2.5)	< 0.001
SRT (ms)	482 (85)	587 (84)	0.02

Data expressed as mean (SD); Type I error was kept at 5% level and two-tailed tests were used.

IOP, intra-ocular pressure in mmHg; MD, mean deviation from standard automated perimetry in decibel (dB); SRT, saccadic reaction time from eye movement perimetry in milliseconds (ms);

*Independent T-test.

### SRT behaviour in superior and inferior GHT sectors in healthy eyes

**Figure 2:**
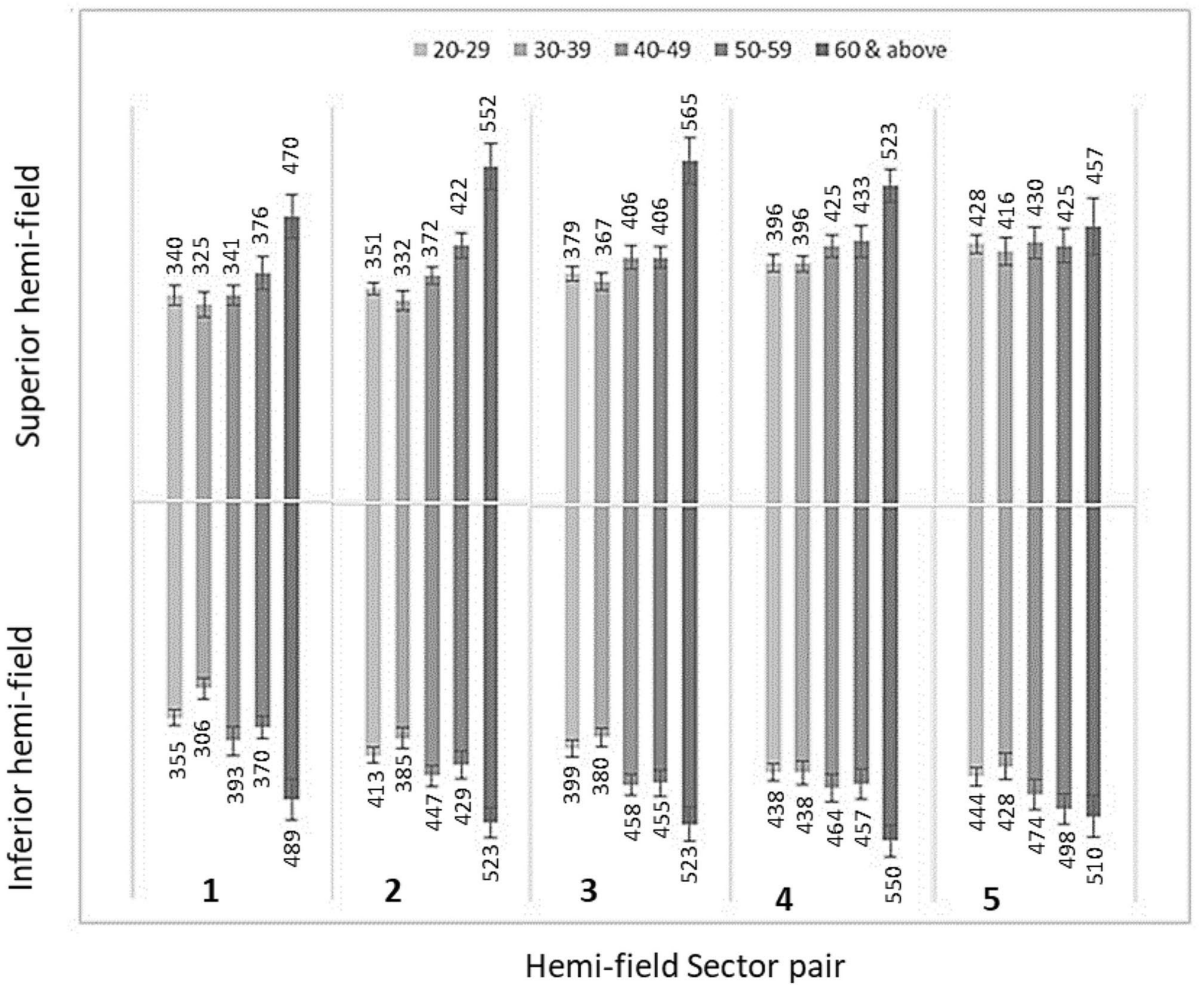
The SRT behaviour in Hemi-field sectors among healthy subjects; The mean SRTs with corresponding standard error was plotted among healthy eyes in different age groups between 20 and 60 years and above for stimulus intensity 214 cd/m^2^. SRT showed symmetrical behaviour across the age groups in eye movement perimetry.

SRTs were compared between superior and inferior sector pairs in all 5 age groups in healthy eyes. Overall, statistically significant (*p* < 0.001) interaction was found between the age groups and visual field sectors. The post-hoc SNK test showed that when compared between the mirror image hemi-field sector pairs (such as hemi-field superior sector 1 and inferior sector 1) the SRTs difference were not statistically significant (*p* > 0.005) for any hemi-field up-down sector pairs. Figure 2 was plotted using the mean SRT responses from healthy eyes in different age groups aged 20–70 for the stimulus intensity 214 cd/m^2^ in the five hemi-field sectors.

### Evaluation of SRT in hemi-field sectors

#### The PSLs in the healthy and glaucomatous eyes

The probability score limits (PSL) scores were assessed in 30 healthy eyes and 30 glaucomatous eyes for each of the 5 hemi-field sector pairs. These per eye scores were compared with the normative PSLs obtained from the phase that dealt with development of hemi-field sectors normative data (Table 2).

### GHT in SAP and PSL in EMP

The Foster et al*.* classification^16^ was used to discriminate eyes into normal and glaucomatous, see Table 5. The sensitivity and specificity was calculated PSL in EMP at all levels using the Foster et al*.* diagnosis as a reference, see Table 6. Since 14 out of 30 of the glaucomatous eyes were misclassified as normal with PSL 0.5%, the sensitivity at this level was compromised to 53% with a promising specificity of 83%. PSL 2.5% showed a balanced sensitivity 70% and specificity 77%.

**Table 5:** Contingency table for PSL from eye movement perimetry using the Foster et al*.*^16^ classification for glaucoma as reference.

Foster et al*.* classification		
Probability score limit in eye movement perimetry	Glaucoma^a^	Normal^a^
PSL_5%		
Glaucoma	21	10
Normal	09	20
PSL_2.5%		
Glaucoma	21	07
Normal	09	23
PSL_1%		
Glaucoma	18	06
Normal	12	24
PSL_0.5%		
Glaucoma	16	05
Normal	14	25

PSL, probability score limits.

^**a**^All values represent number of subjects.

**Table 6:** Sensitivity and specificity with positive and negative predictive values of PSLs based on SRT at different levels.

SRT based PSL	Sensitivity (%)	Specificity (%)	Positive predictive value (%)	Negative predictive value (%)
PSL_5%	70	67	68	69
PSL_2.5%	70	77	75	72
PSL_1%	60	80	75	67
PSL_0.5%	53	83	76	64

PSL, probability score limits; SRT, saccadic reaction time.

**Figure 3:**
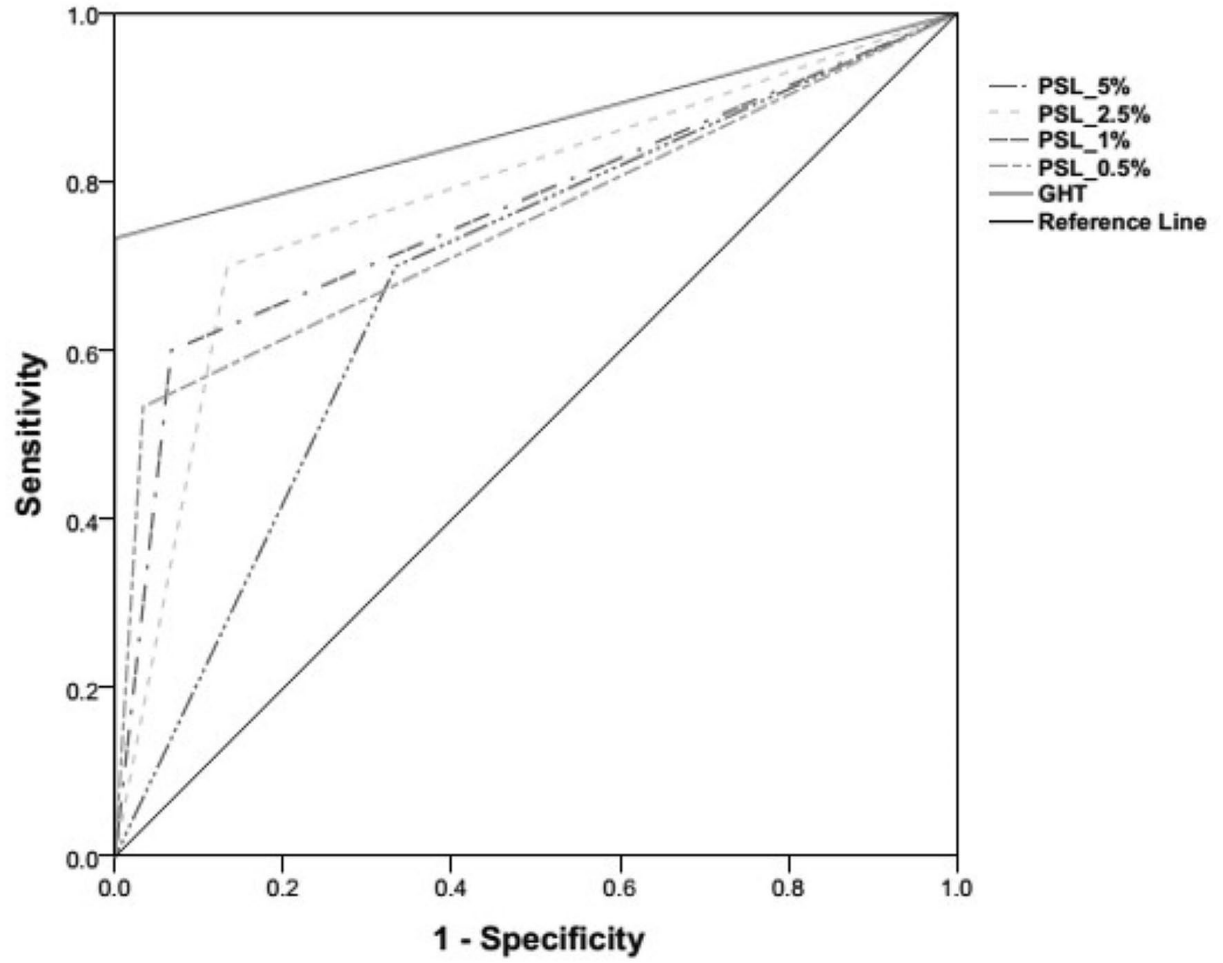
ROC curves plotted for the different levels of the normative PSLs of EMP; The PSL values were obtained from SRT estimated at the level of 5%, 2.5%, 1% and 0.5%.

The area under the ROC curves was plotted for the normative PSLs of EMP at different levels against the Foster et al*.* classification of glaucoma diagnosis, Fig. 3. The Area under the curve (AUC) for the PSLs at different level was ranging between 0.783 and 0.683, shown in Table 7.

**Table 7:** Area under the curve for the PSLs at the level of 5%, 2.5%, 1%, and 0.5%

Variables	Area under the curve*
PSL_5%	0.683
PSL_2.5%	0.783
PSL_1%	0.767
PSL_0.5%	0.750

PSL, probability score limit in eye movement perimetry.

*p value < 0.001.

**Figure 4:**
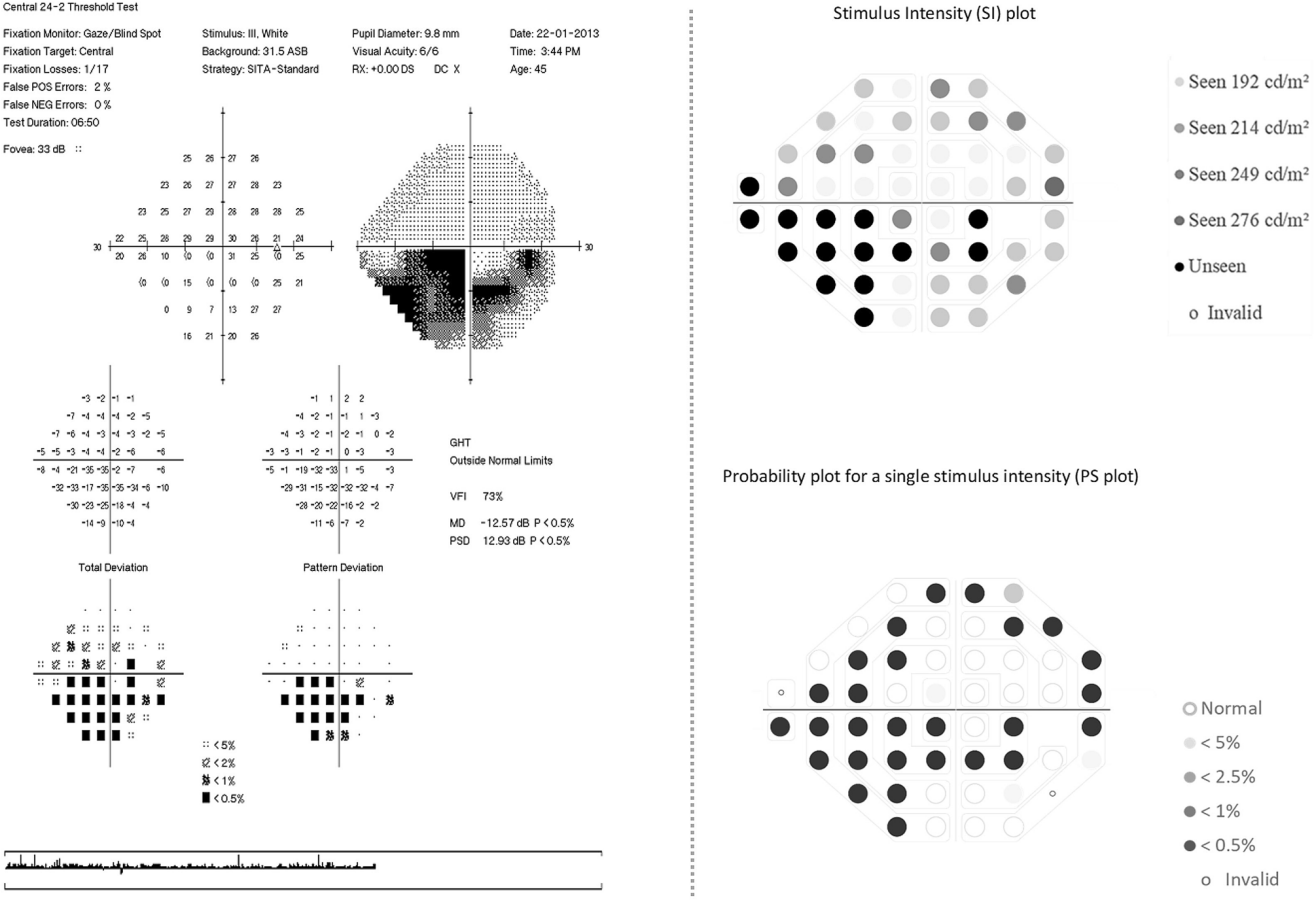
Illustration of HFA and EMP visual field report of a patient with mild glaucomatous visual field defect; Left panel: presentation of HFA 24-2 SITA-standard visual field report identified with mild glaucoma showing shallow nasal defect. HFA indicates Humphrey visual field analyser; SITA, Swedish Interactive Threshold Algorithm. Right panel: the EMP plots for the same patient in 4, left panel, Right top panel presenting saccadic reaction time (SRT) plot at 54 points with corresponding numerical grey scale; Right bottom panel presenting probability plot corresponding to the SRT responses at the stimulus intensity 214 cd/m^2^ presents visual field defect nasally with additional scattered areas of delayed SRT. EMP indicates eye movement perimetry; HFA, Humphrey field analyzer; SITA, Swedish interactive threshold algorithm.

Figure 4, left panel presents a SAP visual field report of a mild glaucoma patient as identified with the clinical evaluation (Foster et al*.* and HAP classification) and GHT showing ‘outside normal limit’. The PSL of this patient obtained in EMP also showed an asymmetry across the hemi-field meridian when compared to the normative PSL and was labelled as ‘abnormal’ Fig. 4, right panel.

**Figure 5:**
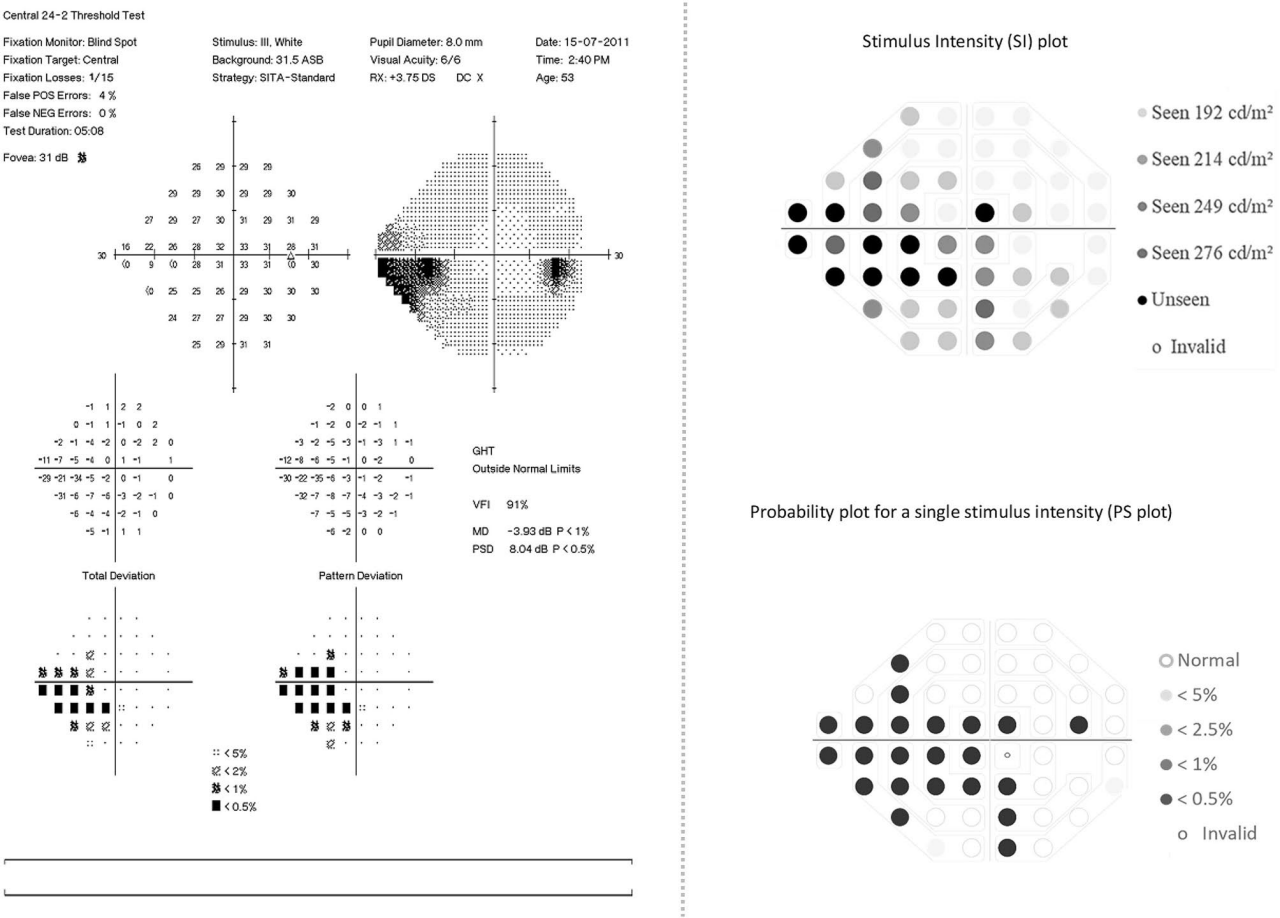
Illustration of HFA and EMP visual field report for a moderate glaucoma patient; Left panel: presentation of HFA 24–2 SITA-standard visual field report showing incomplete inferior arcuate defect. HFA indicates Humphrey visual field analyser; SITA, Swedish Interactive Threshold Algorithm. Right panel: the EMP plots for the same patient in 5, left panel: top panel presenting saccadic reaction time (SRT) plot at 54 points with corresponding numerical grey scale; Bottom panel presenting probability plot corresponding to the SRT responses at the stimulus intensity 214 cd/m^2^ presents visual field defect inferiorly with additional areas of delayed SRT superiorly. EMP indicates eye movement perimetry**;** HFA, Humphrey field analyzer; SITA, Swedish interactive threshold algorithm.

Next, a SAP report of a moderate glaucoma patient is shown in Fig. 5, Left panel with an incomplete inferior arcuate defect and GHT indicating ‘outside normal limit’. Figure 5, Right panel presenting the EMP reports with comparable affected areas as detected with SAP, but here the PSL was labelled as ‘normal’ due to symmetry across the hemi-field meridian, thus the opposite of GHT in SAP.

The visual fields were further classified into three groups normal (n = 30) and a combination of mild (n = 15) and moderate (n = 15) glaucomatous eyes using the HAP classification^17^. To estimate the diagnostic accuracy separately in mild and moderate glaucoma groups, they were compared between the HAP criteria of Humphrey field analyser (HFA) and PSL outcome of EMP. The comparison was done based on the PSL outcome ‘normal’ or ‘abnormal’, where 12 out of 15 mild (at PSL 2.5% and 5%) and 9 moderate (at PSL 2.5%) glaucoma patients were presented with abnormal PSL outcome. The sensitivity and specificity along with positive and negative predictive values presented in Table 8 were calculated for the PSL levels using HAP classification as the reference standard. The PSL at the level of 2.5% showed sensitivity of 67% and 60% in detecting mild and moderate defects respectively (Table 8).

**Table 8:** Diagnostic ability of PSLs from eye movement perimetry in mild and moderate glaucoma using the HAP classification^17^ as reference.

Mild glaucoma (n = 15)	Sensitivity (%)	Specificity (%)	Positive predictive value (%)	Negative predictive value (%)
SRT based PSL				
PSL_5%	80	67	55	87
PSL_2.5%	67	77	59	82
PSL_1%	67	80	63	83
PSL_0.5%	60	83	75	87

Moderate glaucoma (n = 15)	Sensitivity (%)	Specificity (%)	Positive predictive value (%)	Negative predictive value (%)
SRT based PSL				
PSL_5%	60	67	47	73
PSL_2.5%	60	77	56	79
PSL_1%	53	80	57	77
PSL_0.5%	47	83	58	76

*HAP classification: Hodapp Parish and Anderson classification^17^.

## Discussion

The current study investigated the SRT behaviour within the paired sectors of glaucomatous hemi-fields. Since the GHT approach was used with the aim to detect the localised functional loss commonly found in glaucomatous visual field defects, we used the same GHT sectors (Fig. 1) as reported by Asman and Heijl^15^. Here, SRTs showed symmetrical behaviour across superior-inferior hemi-field sectors of GHT when evaluated in healthy eyes. A contrasting pattern was observed in eyes with glaucomatous visual field defects indicating presence of SRT variability between hemi-field sectors.

Sommer and co-workers, reported the first approach of estimating the up-down threshold differences between the mirror image sectors with normative limits^14^. This approach was refined by Asman and Heijl^15^, where they introduced the use of significant deviations from normal limits in the mirror image sector differences instead of actual threshold values. Asman and Heijl’s approach of estimating normal limits showed improved sensitivity and specificity with inclusion of only healthy eyes^15,19^. In the present study, we estimated the normal limits of probability scores (PS) in healthy eyes. Since the effect of age and stimulus eccentricity on SRT in EMP is well documented in the literature^3^, for this study we have derived probability score limits (PSLs) from the significant deviations in location specific SRT from age-corrected normals than actual SRT.

To estimate the classification accuracy on the basis of EMP, we recruited healthy subjects and patients with mild to moderate glaucoma based on the Foster et al*.*^16^ and HAP^17^ classification and excluded those with advanced/severe visual field loss. Inclusion of advanced glaucomatous eyes would likely to have produced little or no asymmetry in the hemi-filed sectors while using SRTs as a measure of visual field responsiveness. However, as suggested by Asman and Heijl, inclusion of such advanced cases would possibly have lowered the hemi-field test sensitivity^15^. This could be explained by the fact that the subjects with advanced visual field loss are expected to have a few ‘seen’ test points and it is often impossible to separate the localised defects from the little or no remaining field of vision.

We decided to restrict our classification of visual field as ‘normal’ and ‘abnormal’ on the basis of PSLs derived from SRT. In the current study, the classification ability for EMP was evaluated on the basis of the PSL asymmetry across the hemi-field meridian only. The specificity ranged between 83 and 67% and sensitivity 70–53% at all the four levels of PSLs [5%, 2.5%, 1%, and 0.5%]. The PSL at the level of 2.5% presented with the optimum combination of specificity 77% and sensitivity 70%, AUC 0.78 (Fig. 3, Table 7) with good positive predictive value—75% and negative predictive value—72% (Table 6). Even though the PSL approach for classifying normal versus abnormal exhibited well enough diagnostic accuracy, it lacked the sensitivity when estimated separately for mild and moderate glaucoma. Here, the sensitivity dropped especially for the moderate cases [sensitivity 60% and specificity 77%, at PSL 2.5%] (Table 8). In mild glaucoma, PSL at 2.5% showed comparatively good discriminatory ability [sensitivity 67% and specificity 77%] than rest of the PSL levels.

The relatively lower sensitivity obtained on the basis of PSL, especially in moderate group, may be due to the fact that SRT tends to show generalised delay throughout the complete glaucomatous visual field. In several other approaches, EMP has shown good ability to discriminate between normal and glaucoma when based on the average delay in SRT^10–13^ and also on the basis of the binary responses, i.e. seen or unseen^4,20^. However, for the current study the magnitude of SRT asymmetry in the mirrored sectors seemed not prominent enough when compared to the light sensitivity threshold in SAP. This is different in GHT. It is worth emphasizing that the approach of GHT was introduced to incorporate the information of retinal nerve fibre layer to enable the possibilities of differentiating localised field loss typical of glaucoma from the generalised one. Najjar et al. and Lamirel et al. reported marked disruptions in SEM in glaucoma patients who exhibited no detectable visual field loss on SAP (pre-perimetric glaucoma)^9,21^. Additionally, another SEM parameter termed as saccadic gain is an important measure in order to describe saccade performance with regard to its accuracy and precision. Saccadic gain is calculated as the ratio between the amplitude of the saccade and the distance of the peripheral target from the fixation target. Lamirel et al. reported that saccadic gain was lower in glaucoma when compared with age-matched healthy subjects^9^. These abnormal eye movements were attributed to the altered neural signalling leading to the inhibition of reflexive saccades. The inclusion of saccadic gain combined with SRT in EMP could be effective in early diagnosis of disrupted eye movement behaviour in glaucoma. In alternative methods of comparing the glaucomatous visual field defects between SAP and EMP based on SRT, delays in SRT tended to produce visual field reports depressed to a higher degree than that of SAP^13^ which is consistent with the findings of McTrusty et al.^20^ based on the ‘seen or unseen’ responses using Saccadic Vector Optokinetic Perimeter. Apparently, these delays are not of localised in nature. Even after adjusting SRT for age and other factors in the visual field, i.e. eccentricity and stimulus intensity and exclusion of patients with any visible ocular media opacities, it still depicting an increase in SRTs in the overall glaucomatous visual fields. We conclude that the classification of normal versus abnormal based on PSL in EMP is limited.

## Conclusion

The current study demonstrates moderate sensitivity and specificity for PSL at 2.5% in detecting abnormal visual fields using Foster et al*.*^16^ and HAP^17^ classification as reference standard. The present data suggest an overall delay in SRT in the glaucomatous visual field. Further clarity on the pathophysiological delay in SRT for patients with glaucoma will aid in explaining the visual field defect pattern to be expected using EMP.

## Data Availability

The datasets generated during and/or analysed during the current study are available from the corresponding author on reasonable request.
